# Multi-Functionalized Heteroduplex Antisense Oligonucleotides for Targeted Intracellular Delivery and Gene Silencing in HeLa Cells

**DOI:** 10.3390/biomedicines10092096

**Published:** 2022-08-27

**Authors:** Mauro Sousa de Almeida, Barbara Rothen-Rutishauser, Michael Mayer, Maria Taskova

**Affiliations:** 1 BioNanomaterials, Adolphe Merkle Institute, University of Fribourg, Chemin des Verdiers 4, 1700 Fribourg, Switzerland; 2 Biophysics, Adolphe Merkle Institute, University of Fribourg, Chemin des Verdiers 4, 1700 Fribourg, Switzerland

**Keywords:** oligonucleotide therapeutics, delivery, modified oligonucleotides, multi-functionalization, gene knockdown

## Abstract

Oligonucleotide therapeutics, antisense oligonucleotides (ASOs) and short interfering RNA (siRNA) are short synthetic nucleic acid molecules with a promising potential to treat a wide range of diseases. Despite considerable progress in the field, the development of safe and effective delivery systems that target organs and tissues other than the liver is challenging. While keeping possible off-target oligonucleotide interactions and toxicity related to chemical modifications in mind, innovative solutions for targeted intracellular delivery are highly needed. Herein, we report on the design, synthesis and testing of a novel multi-modified and multi-functionalized heteroduplex oligonucleotide (HDO) with respect to its intracellular delivery and its ability to silence genes in HeLa cells. Simultaneously, folic acid- and peptide- labeled HDO show proficient silencing of the green fluorescent protein (GFP) gene with an 84% reduction in the GFP fluorescence. In addition, the Bcl2 HDO achieved effective Bcl2 gene knockdown in the cells. The data show the proficiency of the multi-functionalization strategy and provide an example for advancing the design of safe and efficient forthcoming oligonucleotide therapeutics, such as HDO.

## 1. Introduction

Nucleic-acid-based therapeutics have a high potential to treat many terminal diseases caused by genetic defects [1]. Their power dwells in their adaptability towards the target. For instance, a rationally designed and developed oligonucleotide therapeutic sequences that work towards one target gene can be adapted to interfere with a new target gene by changing the nucleotides within the sequence. Compared with traditional small-molecule drugs, oligonucleotide therapeutics are different with respect to the size, the chemical structure and the mode of action. The chemical structure of the small-molecule drug determines the target specificity, the tissue distribution and the metabolism. In the case of oligonucleotide-based therapies, the sequence directs target recognition and the chemistry of the backbone and the vehicle determines tissue distribution and metabolism [2]. 

There are currently two main types of oligonucleotide therapeutics, antisense oligonucleotides (ASOs) and short interfering oligonucleotides (siRNA). While ASOs are single-stranded (4–10 kDa) and siRNA are double-stranded (approx. 14 kDa), these oligonucleotide therapeutics also have different intracellular modes of action. ASOs can exert their action in the cytoplasm and in the nuclei by taking advantage of RNase H enzyme to cut the hybridized target mRNA or by simple steric blocking [3]. On the other hand, siRNA is only active in the cytoplasm and employs the RNA-induced silencing complex (RISC) to perform its action [4]. 

In the past years, both types of nucleic-acid-based therapeutics have been approved by the federal agencies for drug approval [5]. In particular, from 2013 until 2021, seven ASOs and four siRNA oligonucleotide therapeutics were approved by the United States Food and Drug Administration (FDA) and the European Medicines Agency (EMA) [1]. These drugs are targeting rare, previously untreatable diseases, including Duchenne muscular dystrophy, transthyretin-mediated amyloidosis, spinal muscular atrophy and some common conditions, such as hypercholesterolemia [1]. 

However, natural oligonucleotide sequences, having a negatively charged backbones and being susceptible to enzymatic and chemical degradation, lack drug-like properties. The stability of various modified oligonucleotide sequences in bio-fluids and human serum is studied and described [6,7,8]. In order to overcome the challenges of possible off-target effects combined with improved chemical and enzymatic stability, previous work has reported many chemical modification strategies of the backbone and nucleotides. The approved ASOs usually contain a phosphorothioate (PS) backbone (e.g., the drug Mipomersen) where one non-binding oxygen is replaced by a sulfur, [9], 2’-O-(2-methoxyethyl) (2’-MOE) nucleotide modification (e.g., the drugs: Inotersen, Nusinersen and Volanesorsen) [10,11], or phosphorodiamidate-morpholino (PMO) units (e.g., the drugs Eteplirsen, Golodirsen and Casimersen) that replace the natural sugar-phosphate backbone [12,13,14]. On the other hand, the siRNA therapeutics on the market, including the drugs Givosiran, Lumasiran and Inclisiran, comprise partial PS backbone modification, 2’-O-methyl (2’OMe) and 2’fluoro (2’F) nucleotide modification. In addition, N-acetyl galactosamine dendrimer is covalently attached to the 3’terminus of their sense strand [15,16,17]. Moreover, the structure of the approved drug Patisiran as siRNA includes 2’OMe nucleoside modification and two dT-oligodeoxyribonucleotides overhanging on the 3’ terminus of both the sense and antisense sequences. To achieve intracellular delivery, Patisiran is formulated as a lipid nanoparticle drug using ionizable lipids [18]. 

Recently, Nishina et al. reported efficient gene silencing performed by a novel type of therapeutic oligonucleotides designed as antisense double-stranded, heteroduplex oligonucleotides (HDOs) [19,20]. The authors developed short HDO of DNA and RNA where the DNA antisense strand was designed as a DNA/LNA gapmer while the complementary RNA sense strand was 2’OMe-modified and conjugated with α-tocopherol. The authors reported that this type of HDO was more potent to knockdown the target mRNA compared with the parent single-stranded DNA/LNA ASO [19]. Moreover, Asami et al. reported on an efficient gene suppression by a DNA/DNA double-stranded oligonucleotide in vivo [21]. The authors showed that the ASO/DNA and ASO/RNA HDO have higher potency and efficacy in vivo than the parent single-stranded ASO. In addition, a report by Yoshioka et al. showed improved intracellular potency of an ASO/RNA HDO to silence microRNA in vivo. This candidate could silence the target microRNA with 12-fold higher efficiency than the parent single-stranded ASO [22]. Furthermore, Rodrigues et al. developed a novel way of silencing oligonucleotides called polypurine reverse Hoogsteen hairpins (PPRHs) [23]. The authors designed the PPRHs as DNA formed by two antiparallel polypurine oligonucleotides linked by a five-thymidine loop and reported a decrease in target mRNA levels in prostate cancer cells and decreased tumor volume [24]. The same group using repair-PPRH also reported on a successful correction of different single-point mutations in mammalian cells [25]. 

Despite significant progress in the field of oligonucleotide therapeutics, the biggest issue preventing its widespread use is the low efficiency of intracellular delivery to organs and tissues other than the liver [26]. To date, efficient delivery of oligonucleotide therapeutics is reported through local delivery (intravitreal and intrathecal) [26,27] and to the liver [28]. The delivery strategies include chemical modifications of the nucleotides and the backbone, covalent conjugation with molecules or biomolecules and lipid nanoparticle formulation. While considering the rationally modified sequence design to avoid off-target side effects (i.e., sequence degradation and sequence-related toxicities), there is immense need for new delivery technologies that will facilitate the clinical translation of the oligonucleotide therapeutics.

Herein, we explored the design and synthesis of novel multi-functionalized HDOs for targeted delivery and gene silencing in HeLa cells. We tested the intracellular delivery and the suppression of the target gene activity. 

## 2. Materials and Methods

Modified and functionalized oligonucleotides were ordered from LubioScience GmbH, Switzerland. Folate-Peg3-Azide was ordered from Base click GmbH, Germany. The thiol/azide-modified peptides (P1 and P2) were ordered from Gen Script Biotech. Lipofectamine 3K, RNAiMax and Opti MEM were ordered from Thermo Fisher Scientific. Green-fluorescent-protein (GFP)-expressing HeLa cells were purchased from Amsbio. All other reagents were ordered from Sigma Aldrich, Buchs, Switzerland.

### 2.1. Design Tools and Strategy

We selected a series of single-stranded ASOs (ASO1-ASO6, six in total) that target the open reading frame (ORF) of the green fluorescent protein (GFP) gene (in HeLa cells expressing GFP) manually or used the Sfold software for statistical folding of nucleic acids. The GFP HDO consists of ASO5/ASO5.1/ASO5.2 and its complementary functionalized RNA sequence (ASOss).

**Scheme 1:**
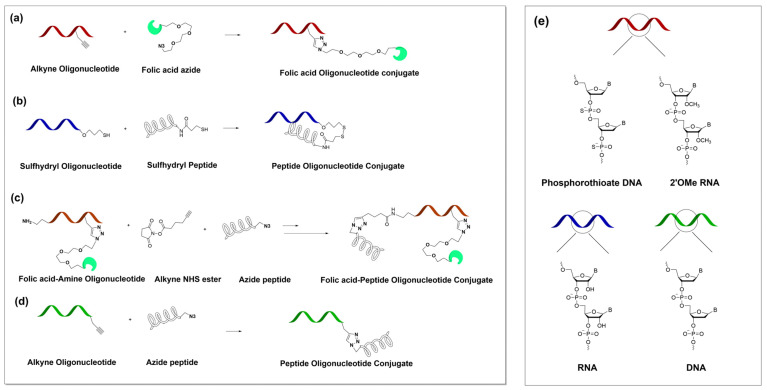
Conjugation strategies used and the chemical structure of the oligonucleotides. (**a**,**d**) copper-catalyzed azide-alkyne cycloaddition (CuAAC) chemistry; (**b**) conjugation by disulfide bond; (**c**) reaction of the amino group with an alkyne NHS ester to install a new alkyne group to the oligonucleotide followed by a CuAAC reaction; and (**e**) antisense oligonucleotide (red) consisting of a phosphorothioate backbone and terminal 2’OMe sugar modifications while the sense oligonucleotide (blue) is a pure RNA and (green) is a pure DNA.

The advanced ASO5 analogue, ASO5.2 was rationally designed as a gapmer with a full phosphorothioate backbone and with three 2’OMe modifications on the two termini. Additionally, we installed an alkyne functionalization at internal position 16 (counting 5’→3’). The ASOss was designed as a pure RNA oligonucleotide functionalized with a thiol group at the 5’ terminus (Scheme 1 and Scheme S1). We also included a scrambled control for ASO5 named ASOscr.

We designed the Bcl2 HDO targeting the *Bcl2* gene using a similar concept. The antisense oligonucleotide, Bcl2 ASO.1 was designed as a full phosphorothioate backbone oligonucleotide with partial three 2’OMe modifications at both termini 5′ and 3′ and an alkyne functionalization at internal position 16 (counting 5′→3′). Additionally, this sequence contains an amino group modifier at the 5′ terminus (Scheme S1). The sense strand, Bcl2ss was designed as a pure DNA sequence and contained alkyne functionalization at the 3’ terminus. Scrambled control Bcl2scr was also included.

### 2.2. Conjugation Using Copper-Catalyzed Azide-Alkyne Cycloaddition (CuAAC) Chemistry

The alkyne-modified ASO5.2/Bcl2 ASO.1 (20 nmol) was mixed with Folate-Peg3-Azide (100 nmol) in Milli-Q (MQ) water/DMSO/t-BuOH (2:1:1) solvent mixture with a total volume of 200 µL. Next, 10 µL of 2M triethylammonium acetate buffer (TEAA), pH = 7 was added. After degassing under argon gas, 10 µL of 10 mM Cu(II)TBTA solution and a freshly prepared solution of 4 µL of 50 mM L-ascorbic acid were added. The resulting reaction mixture was again deaerated under argon, stirred at 40 °C in the dark for 4 h and at rt in the dark for an additional 12 h. Finally, the reaction mixture was desalted and purified on a NAP5 column (GE Healthcare). The obtained solution was dried on a SpeedVac and dissolved in (MQ) water. The concentration of the product was obtained by measuring the absorbance at 260 nm. The product was characterized using IC HPLC (Figure S5A,B,E,F) and Maldi TOF mass spectrometry (Figure S6A,C,D).

The alkyne-modified Bcl2ss (20 nmol) was mixed with Peptide 2-azide, (P2) (20 nmol) in MQ water and 7% formamide with a total volume of 200 µL. Next, 10 µL of 2M triethylammonium acetate buffer (TEAA) and 4 µL of 50 mM aminoguanidine hydrochloride were added. After degassing under argon gas, 10 µL of 10 mM Cu(II)THPTA solution and a freshly prepared solution of 4 µL of 50 mM L-ascorbic acid were added. The resulting reaction mixture was again deaerated under argon and stirred at rt in the dark for 18 h. Finally, the reaction mixture was desalted and purified on a NAP5 column (GE Healthcare). The obtained solution was dried on a SpeedVac and dissolved in MQ water. The concentration of the product was obtained by measuring the absorbance at 260 nm. The product was characterized using IC HPLC (Figure S5G,H) and Maldi TOF mass spectrometry (Figure S6E,F).

The Bcl2 ASO.1-FA-P2 was synthetized by first attaching the FA to the Bcl2 ASO.1 by CuAAC, as described above. Next, we converted the amino group to an alkyne group by reaction with an alkyne NHS ester. In particular, we mixed Bcl2 ASO.1-FA (oligo amine) 20 nmol in 90 µL of 0.2 M PBS buffer, pH = 8.3 with 8 molar excess of alkyne NHS ester in 10 µL DMSO. The resulting mixture was vortexed overnight, desalted and purified using a NAP5 column (GE Healthcare). The obtained solution was dried on a SpeedVac and dissolved in MQ water. The concentration of the product was obtained by measuring the absorbance at 260 nm. The product was characterized using IC HPLC (Figure S5I,J).

### 2.3. Conjugation by Disulfide Bond

To the complementary RNA oligonucleotide thiol-modified, ASOss (20 nm, 20 µL), Tris[2-carboxyethyl] phosphine (TCEP) (2000 nM, 4 µL) and 2M TEAA (5 µL) were added and the resulting mixture was stirred at rt for 4 h. Subsequently, the mixture was desalted and purified on a NAP5 column and dried on a SpeedVac. Next, the sulfhydryl RNA was redissolved in MQ/ACN (4:1) solvent in total volume of 200 µL that also contained 2M TEAA (5 µL). After the addition of 2.2’-dithio-dipyridin (200 nmol), the resulting mixture was stirred at rt for 1 h and directly purified using a NAP5 column and dried on a SpeedVac. Finally, the activated oligonucleotide was dissolved in 20 µL of MQ water, 70 µL of formamide and 5 µL of 2M TEAA. After the addition of the Peptide 1 (P1), the mixture was vortexed at rt for 3 h. The final product was purified using a NAP5 column, dried on a SpeedVac and dissolved in MQ water. The concentration of the product was obtained by measuring the absorbance at 260 nm. The product was characterized using IC HPLC (Figure S5C,D) and Maldi TOF mass spectrometry (Figure S6B).

### 2.4. Cell Culture Studies

Parental HeLa cells and HeLa cells expressing GFP were maintained in Dulbecco’s Modified Eagle’s Medium (DMEM) and a folate-free Roswell Park Memorial Institute (RPMI) medium supplemented with 10% fetal bovine serum (FBS), 1% penicillin-streptomycin (pen-strep) and 2 mM L-Glutamine, kept at 37 °C in a humidified atmosphere with 5% CO_2_. We passaged the cells twice per week.

### 2.5. Carrier-Free Transfection (without Lipofectamine)

For the transfection experiments, HeLa/HeLa GFP cells were first seeded into a 24-well plate at a density of 4.0 × 10^4^ cells per well in 400 µL folate-free RPMI without pen-strep antibiotic. The next day, we added the ASOs/HDO/Scrambled control in Opti-MEM in different concentrations with a total volume of 100 µL to the cells. Accordingly, the knockdown effect of the probes was followed by fluorescence measurement over time or measured by RT-qPCR after 48 h. When applicable, the effect of the probes was also measured by flow cytometry after 48 h.

### 2.6. Control Transfection with Lipofectamine

HeLa and HeLa GFP cells were seeded into a 24-well plate at a density of 4.0 × 10^4^ cells per well in 400 µL of folate-free RPMI that did not contain pen-strep antibiotic. The next day, the ASOs/HDO/Scrambled control was co-transfected using Lipofectamine 3000 following the manufacturer’s protocol. Accordingly, we followed the knockdown effect of the Lipofectamine-probes (lipoplexes) by fluorescence measurements over time or by flow cytometry after 48 h.

### 2.7. Fluorescence Measurement over Time

The mean fluorescence intensity of the GFP-expressing cells was followed and measured on an Incucyte Essen Bioscience instrument at 37 °C in a humidified atmosphere with 5% CO_2_. Scans of a minimum of 4 per well were made every 12 h for a total of 48 h after transfection of the ASOs. The data were analyzed using Incucyte ZOOM 2018A software.

### 2.8. Flow Cytometry

Post transfection/lipofectamine co-transfection (after 48 h), the cells in the wells were washed with 1× PBS followed by detachment from the culture plates (using 50 µL 0.05% trypsin with 0.02% Ethylenediaminetetraacetic acid (EDTA)) and transferred into tubes. After centrifugation (2000 rpm for 5 min) and aspiration of the supernatant cells, the cells were suspended in 200 µL of flow cytometry (FC) buffer (1× PBS that contains 2% FBS and 3 mM EDTA). We performed flow cytometry studies immediately on a FACS Canto II instrument following the manufacturer’s protocol.

### 2.9. RT-qPCR

After transfection, total RNAs were isolated from the cells growing in 24-well plates. Cell lysis was performed directly in the well, using 100 μL of BL + TG buffer (Promega). The lysates from 2 wells were pooled together and total RNA was extracted using ReliaPrep™ RNA Cell Miniprep System (Promega, Z6012, Madison, WI. USA) following the manufacturer’s protocol. The quality and quantity of RNA were analyzed by a Thermo Scientific™ NanoDrop™ 2000 Spectrophotometer and Agilent 2100 Bioanalyzer (Agilent Technologies, Santa Clara, CA, USA). Equal quantities of RNA, from each sample, were used to prepare the complementary DNA (cDNA). The reverse transcriptase reaction was performed with the Omniscript RT system (Qiagen, Germany), OligodT (Microsynth, Switzerland), and RNasin Plus RNase Inhibitor (Promega, Switzerland). The synthesis of cDNA was performed by using 6.5 µL of isolated RNA (250 ng), 1 μL of oligo-dT primer (10 μM), 1 µL of dNTP Mix (5 mM), 0.25 µL of RNase inhibitor, 0.25 µL of Omniscript reverse transcriptase (1 Unit), and 1 µL of buffer RT. The real-time PCR was performed by mixing 2 μL of 3-fold diluted cDNA with 5 μL of SYBR-green master mix (Fast SYBR Green master mix, Applied Biosystems), 2 μL of nuclease-free water (Promega, Madison, WI. USA) and 2 μL of primer mix (180 nM) on a 7500 fast real-time PCR system (Applied Biosystems). Relative expression levels for each gene of interest were calculated using the Pfall method [29], with glyceraldehyde-3-phosphate dehydrogenase (GAPDH) and tyrosine 3-monooxygenase/tryptophan 5-monooxygenase activation protein zeta (YWHAZ) as internal standard genes. Details about the primers are included in the supplementary information (Table S2).

### 2.10. Cell Proliferation Assay

HeLa GFP cells were seeded into a 24-well plate at a density of 4.0 × 10^4^ cells per well in 400 µL of folate-free RPMI without pen-strep antibiotic. The next day, the cells were transfected following the carrier-free protocol (described above) and incubated for an additional 48 h. A cell viability assay was evaluated using the CytoPainter cell proliferation staining reagent from Abcam’s (ab176736) using the manufacturer’s protocol. In short, 48 h post incubation, we washed the cells with 1× PBS, trypsinized and collected the cells into Eppendorf tubes. Next, 1× dye working solution was added and the cells were incubated at 37 °C for 30 min. After removal of the dye working solution, we washed the cells with the provided buffer and prepared them for flow cytometry as described above. For the measurements, we used a red laser (633 nm) and APC filter. We analyzed the flow cytometry data using the FlowJo software v.10.

### 2.11. Relative Expression of the Folate-Binding Protein on the Cell Surface of HeLa and HeLa GFP Cells

For this assay we used APC anti-human Folate Receptors alpha and beta and APC Rat IgG2a, kappa Isotype Ctrl antibodies from BioLegend. HeLa/HeLa GFP cells (0.5 million per condition) were counted, placed in Eppendorf tubes and centrifuged at 2000 rpm for 10 min at 4 °C. Next, the medium was aspired and the cells were suspended in 50 µL of staining buffer (BioLegend). Antibodies were added to each cell suspension (5 µL per 1 million cells) and the cells were incubated in the dark, on ice for 30 min. Next, the cells were centrifuged at 2000 rpm for 5 min at 4 °C followed by aspiration of the supernatant and washing with 1 mL staining buffer. The washing and centrifugation steps were repeated one more time and finally the cells were suspended in flow cytometry buffer. For the measurements, we used a red laser (633 nm) and APC filter. We analyzed the flow cytometry data using the FlowJo software v.10.

### 2.12. Expression of the Folate-Binding Protein on the HeLa Cells Surface

We determined the surface expression of the folate receptor in GFP HeLa cells following the previously described method [30]. Cells were detached using trypsin, washed twice with PBS and suspended in a staining buffer (BioLegend). For the binding assay, we incubated a fixed number of 0.5 million cells per condition with increasing concentrations of antibody, APC anti-human Folate Receptors alpha and beta (0–0.5 µg) for 30 min on ice. For the depletion assay, we incubated an increasing number of cells (0.5–4 million) per condition with a fixed concentration (0.1 µg) of antibody, APC anti-human Folate Receptors alpha and beta for 30 min on ice. Next, the cells were centrifuged at 2000 rpm for 5 min at 4 °C followed by aspiration of the supernatant and washing with 1 mL of staining buffer. The washing and centrifugation steps were repeated one more time and finally the cells were suspended in flow cytometry buffer. For the measurements, we used a red laser (633 nm) and APC filter. We analyzed the flow cytometry data using the FlowJo software v.10.

## 3. Results

### 3.1. Conjugation and Preparation of Multi-Modified Heteroduplex Oligonucleotides (HDOs)

We designed the GFP HDO with the aim to target the GFP gene in the GFP-expressing HeLa cells consisting of ASO5.2 as an antisense strand and ASOss as a sense strand. The advanced oligonucleotide sequence ASO5.2 contains phosphorothioate backbone modifications, terminal 2’OMe sugar modifications and internal functionalization with an alkyne group. We included the backbone and the sugar modifications to stabilize the oligonucleotide against enzymatic degradation. Moreover, we used an internal alkyne functionalization to conjugate the ASO5.2 with folate–azide (Scheme 1). The sense strand ASOss was designed as pure RNA to contain a 3’terminal thiol modification and was conjugated to the thiol-peptide 1 (P1) by a disulfide bond (Scheme 1). We constructed the resulting multi-modified GFP HDO-FA-P1 by hybridization of ASO5.2-FA with ASOss-P1 (Scheme S1).

Next, the Bcl2 HDO for targeting the Bcl2 gene comprised of the antisense strand Bcl2 ASO.1 and the sense strand Bcl2ss. The Bcl2 ASO.1 follows a similar design to the ASO5.2 in regards to the modifications described above with an additional amino modifier at the 5’ terminus. We used internal alkyne functionalization to conjugate the Bcl2 ASO.1 with folate–azide. In addition, as a separate single-stranded probe we further converted the terminal amino modification to an alkyne group and conjugated it with azide-peptide 2 (P2) as described above. We designed the sense strand Bcl2ss as pure DNA to contain alkyne functionalization at the 3’ termini and conjugated it with azide P2 as described above. We created the resulting multi-modified Bcl2 HDO-FA-P2 by the hybridization of both antisense (Bcl2 ASO.1-FA) and sense (Bcl2ss-P2) oligonucleotides (Scheme S1).

The peptide sequences P1 and P2 (SI Table S1), both consisted of 14 amino acids and contained the same amino acid sequence. One difference is that P1 contained a terminal 3-mercaptopropyl while P2 contained terminal azide functionality.

### 3.2. Relative Expression of the Folate-Binding Protein in HeLa Cells

**Figure 1:**
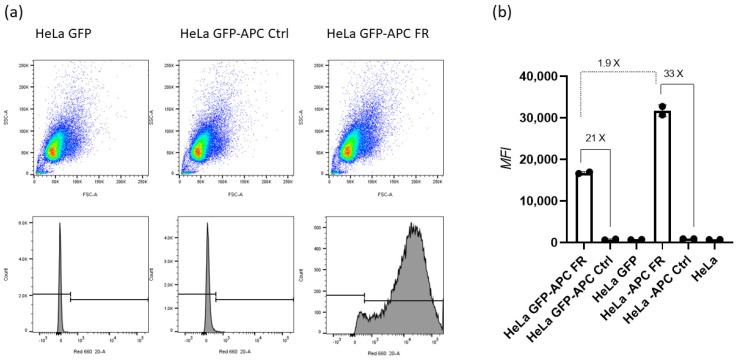
Relative expression levels of cell-surface folate-binding proteins (receptor) in HeLa and HeLa GFP cells expressing green fluorescent protein (GFP). (**a**) determined by flow cytometry; (**b**) median fluorescence intensity; APC FR represents allophycocyanin-labeled antibody against anti-human Folate Receptors alpha and beta, while APC Ctrl represents a allophycocyanin labeled Rat IgG2a, Ctrl antibody (kappa isotype).

We determined the relative expression of the folate receptor in HeLa and GFP-expressing HeLa cells. Figure 1 shows that allophycocyanin (APC)-labeled anti-human folate receptor (FR) antibody displayed a 21-fold increase in folate receptor binding compared with APC-labeled Ctrl antibodies in HeLa GFP cells. This effect was even higher in the parental HeLa cells with APC-labeled anti-FR antibody showing 33-times higher folate receptor binding compared with APC-labeled Ctrl antibodies.

In addition, as described in the SI, we determined the average number of folate receptor per GFP HeLa cell using titration data (Figure S4). We found that on average each HeLa cell expresses 1.7 million folate-binding protein molecules.

### 3.3. GFP Gene Silencing in GFP-Expressing HeLa Cells by Multi-Modified GFP HDO

**Figure 2:**
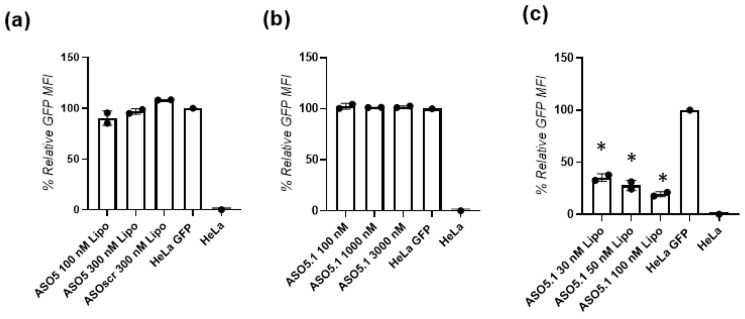
Relative GFP fluorescence in GFP-expressing HeLa cells. Transfection was performed (**a,c**) with lipofectamine (Lipo) or (**b**) without. GFP expression was assessed measuring the median fluorescence intensity (MFI) 48 h post transfection with the ASOs by flow cytometry. Error bars indicate standard deviation (SD) of at least two independent biological replicates. * *p* < 0.05 versus HeLa GFP calculated by one-way ANOVA.

Prior to investigating the GFP HDOs, we tested the parent ASOs for their ability to perform gene silencing after carrier-free transfection or transfection with lipofectamine reagent (Lipo) in the GFP-expressing HeLa cells. We observed that all of the natural DNA ASOs (ASO1–ASO6), even when transfected with Lipo, performed poorly in regards to GFP gene silencing (SI Figure S2). Since ASO5 showed minimal activity (Figure 2a), we continued to modify it and designed an advanced ASO5.1 sequence that contained a phosphorothioate backbone and 2’OMe modified termini. Figure 2b,c shows that when ASO5.1 was transfected carrier-free (without Lipo) in three different concentrations (100 nM, 1000 nM and 3000 nM) it was not able to silence the GFP gene. However, when the same oligonucleotide was transfected with Lipo, it efficient silence the gene in a concentration-dependent fashion. In particular, ASO5.1 could reduce the GFP fluorescence by 65%, 72% and 80% when transfected with Lipo in 30 nM, 50 nM and 100 nM concentrations, respectively. As expected, the ASOscr (scramble control) showed no gene knockdown activity.

**Figure 3:**
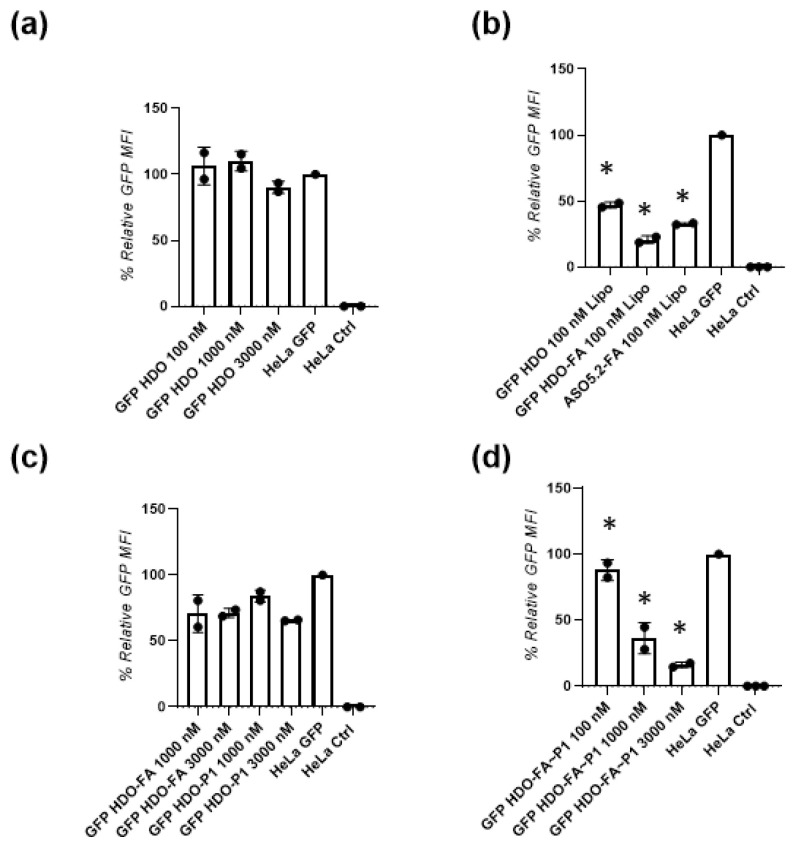
Relative GFP fluorescence in GFP-expressing HeLa cells. Transfection was made (**b**) with lipofectamine (Lipo) * *p* < 0.05 or (**a**,**c**,**d**) * *p* < 0.05 without. HDO is a heteroduplex oligonucleotide; FA is folic acid; P1 is peptide 1. GFP expression was assessed measuring the median fluorescence intensity (MFI) 48 h post transfection on an IncuCyte cell-imaging microscope. Error bars indicate standard deviation (SD) of at least two independent biological (cell culture) replicates.

Next, we investigated the gene-silencing ability of the GFP HDO that consisted of ASO5.1 and ASOss not conjugated to folic acid or P1. Figure 3a shows that the GFP HDO used at 100 nM and 1000 nM concentrations failed to reduce GFP gene expression. Nevertheless, when GFP HDO was transfected at 3000 nM concentration, it showed a minimal (10%) reduction in GFP fluorescence. When the same GFP HDO was transfected with Lipo, it showed downregulation of the GFP gene by a reduction in the fluorescence by 53%. To confirm that the improved design of the ASO5, modified and conjugated with folic acid (ASO5.2-FA), could downregulate the GFP gene, we tested the same molecule using transfection with Lipofectamine. The ASO5.2-FA Lipo displayed a 64% reduction in the GFP fluorescence 48 h post Lipo transfection (Figure 3b). Moreover, when the GFP HDO was conjugated with folic acid (GFP HDO-FA) or, more precisely, the ASO5.1 was conjugated with FA and hybridized to ASOss to form GFP HDO-FA and transfected with Lipo, it performed efficient GFP gene silencing with an 80% reduction in GFP fluorescence (Figure 3b). Next, we assessed the ability of GFP HDO-FA and the GFP HDO-P1 to silence genes without Lipo. Notably, as seen in Figure 3c, the GFP HDO conjugated only with folic acid displayed an approximately 30% reduction in the GFP fluorescence independent from the concentration used (1000 nM and 3000 nM). On the other hand, the GFP HDO conjugated with P1 only showed a 15% reduction in the GFP fluorescence when used in 1000 nM and a 35% reduction after treatment with 3000 nM.

Finally, to examine the multi-modified GFP HDO-FA-P1 that consisted of hybridized ASO5.2-FA with covalently attached folic acid and ASOss conjugated with P1, we performed carrier-free (without Lipo) transfection in three different concentrations (Figure 3d). In contrast to the non-conjugated GFP HDO, the GFP HDO-FA~P1 displayed proficient, concentration-dependent GFP gene silencing with a reduction in the GFP fluorescence by 12%, 64% and 84% when used in 100 nM, 1000 nM and 3000 nM concentration, respectively.

When we investigated the gene silencing ability of the antisense ASO5.2-FA, conjugated with folic acid in 100 nM and 1000 nM concentrations, no effect was observed. Additionally, prior transfection we complexed the ASO5.2-FA with P1 resulting in ASO5.2-FA/P1 by simple mixing followed by an incubation period. The data (SI Figure S3) show an insignificant gene silencing of <10% after treatment with 100 nM ASO5.2-FA/P1 and no effect after treatment with 1000 nM. As a control, we also tested the sense ASOss-P1 conjugated with P1 and, as expected, a reduction in the GFP fluorescence was absent (SI, Figure S3).

### 3.4. Cell Viability after Treatment with Heteroduplex ASO

We assessed the cell viability by a CytoPainter cell proliferation assay after the transfection of the GFP HDO-FA-P1 in three different concentrations of 100 nM, 1000 nM and 3000 nM and transfection of the GFP HDO-FA-P1 with Lipo using 100 nM concentrations (SI, Figure S1). In all cases, the cell viability remained high, similar to the non-treated GPF-expressing HeLa and normal HeLa cells. The cell proliferation was slightly affected (94% viability) when the GFP-expressing HeLa cells were treated with 100 nM GFP HDO-FA-P1 complexed with Lipofectamine (SI, Figure S1).

### 3.5. Bcl2 Gene Silencing by the Multi-Modified Bcl2 HDO

**Figure 4:**
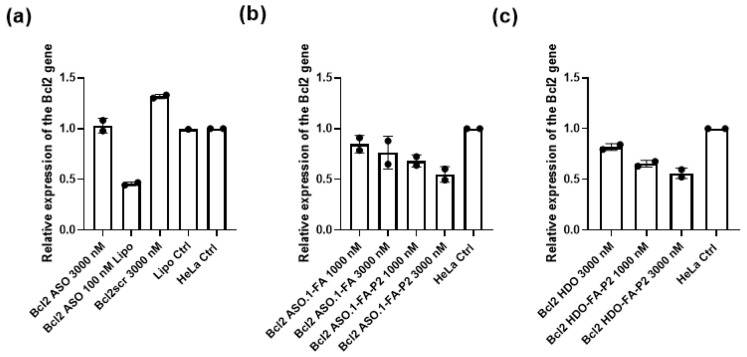
Normalized *Bcl2* gene expression in HeLa cells 48 h after transfection (**a**) with, (**b,c**) without lipofectamine (Lipo)). HDO is heteroduplex oligonucleotide, FA is folic acid and P2 is peptide 2. Lipo Ctrl is the control experiment where we treated the cells with lipofectamine solution without oligonucleotide added. The data were normalized using the *GAPDH* and the *YWHAZ* genes as reference genes. Error bars indicate standard deviation (SD) of at least two independent biological replicates.

We first tested the *Bcl2* gene silencing activity of the Bcl2 ASO using 3000 nM concentration. The advanced modified Bcl2 ASO without conjugation with FA displayed no activity of *Bcl2* gene knockdown (Figure 4a). As expected, the same was true for the scrambled control (Bcl2scr used in 3000 nM concentrations). When we transfected the Bcl2 ASO using Lipo, it showed by 54% *Bcl2* gene knockdown (Figure 4a). Next, we hybridized and tested the Bcl2 HDO that consisted of Bcl2 ASO and Bcl2ss (without conjugated biomolecules). This probe used in high 3000 nM concentrations showed by 19% *Bcl2* gene knockdown (Figure 4c). It is noteworthy that the Bcl2 HDO-FA-P2 that contained both hybridized molecules, namely Bcl2 ASO.1-FA (conjugated with folic acid) and the Bcl2ss (conjugated with P2) showed a higher gene-silencing efficacy. Bcl2 HDO-FA-P2 displayed by 35% and by 44% silencing after treatment with 1000 nM and 3000 nM concentrations, respectively (Figure 4c).

Finally, we also examined the single-stranded multi-modified antisense oligonucleotide Bcl2 ASO.1 conjugated just with folic acid (Bcl2 ASO.1-FA) or conjugated with both, folic acid and P2 (Bcl2 ASO-FA-P2). The Bcl2 ASO-FA, internally modified with folic acid showed by 16% gene knockdown when used in 1000 nM concentrations and by 24% gene knockdown when used in 3000 nM concentrations (Figure 4b). However, Bcl2 ASO-FA-P2, the same antisense strand additionally conjugated with the P2 on the termini, displayed higher efficacy. The gene knockdown ability of the Bcl2 ASO-FA-P2 was concentration-dependent and, in particular, was by 32% and by 46% using 1000 nM and 3000 nM concentrations, respectively (Figure 4b).

## 4. Discussion

Herein we investigated the ability for targeted intracellular delivery of multi-modified and multi-functionalized HDOs. These advanced HDOs consisted of a modified antisense strand (ASO) that bore a triazole internally linked folic acid and a pure RNA/DNA sense strand decorated with a short peptide sequence on the 3’ termini. We have shown that the HDO conjugated with folic acid and peptide can efficiently silence GFP and *Bcl2* genes in GFP-expressing and wild-type HeLa cells, respectively.

We first rationally designed the oligonucleotides forming the HDO and selected the ligand and the peptide sequence. For the GFP HDO, the ASO was chemically modified to contain phosphorothioate instead of a natural phosphodiester backbone and terminal 2’OMe sugar modifications on both termini, 3′ and 5′, giving the possibility for both RNase H or steric blocking mechanisms of action. Previous reports show that phosphorothioate-modified oligonucleotides possess higher resistance towards extra- and intracellular degradation by nucleases and assist cellular uptake and bioavailability in vivo [31]. Further, Hoon Yoo et al. demonstrated that 2’OMe sugar modifications do not show toxic effects and they are resistant to serum nucleases [32]. Moreover, the ASO bears an internal triazole-linked folic acid. We chose folic acid as a specific ligand to target the folate receptor expressed at the HeLa cell surface. Fernandez et al. reported that the folate receptor was 100–300 times overexpressed (on the order of 1–10 million receptor copies per cell) in ovarian cancer compared with normal cells [33]. Our results of approximately 1.7 million receptor copies per HeLa cell are in this order of magnitude and confirm the sufficient expression of the folate receptor to be used for targeted delivery by the folic acid ligand. We used CuAAC chemistry to attach the folic acid azide to the ASO alkyne because of its selectivity and ability to give products in high purity and yields [34,35]. In regards to ASOss, we designed it as a pure RNA sequence conjugated with P1 via a disulfide bond [36,37]. The rationale behind this choice is that the disulfide bond can be reduced in the endosomes and the peptide can thereby detach from the GFP HDO [38].

We designed the Bcl2 ASO.1 representing the active part of the Bcl2 HDO to follow the same structural modifications and folic acid label as the ASO in GFP HDO. However, the Bcl2 ASO.1 contains an additional amino functionality on the 5’terminus that opens the possibility of an additional attachment of P2 by CuAAC chemistry. On the other hand, we designed the Bcl2ss as a pure DNA sequence and conjugated it via CuAAC chemistry to P2.

First, we assessed the parent ASOs (pure DNA sequences, ASO1–ASO6) for their ability to perform GFP gene silencing in GFP-expressing HeLa cells. The data show insignificant gene silencing even when transfected with Lipo. Since ASO5 presented a minor gene silencing effect (10%), we upgraded it to a modified sequence named ASO5.1. While the ASO5.1 with high 3000 nM carrier-free transfection failed to show biological activity, the same sequence, when transfected with Lipo in 100 nM concentrations, showed efficient gene silencing by 80%. These results confirm that while the advanced modified version sequence -ASO5.1 is capable for proficient GFP gene knockdown, it still lacks the ability of intracellular delivery without the transfection agent Lipo. This observation is in agreement with previous reports confirming that in contrast to natural DNA and RNA sequences, modified oligonucleotides are resistant to enzymatic degradation and can stay intact in biological media to perform gene knockdown [7].

Next, we show that the GFP HDO (not conjugated with the biomolecules) when transfected with Lipo, showed efficient gene silencing by 53%. However, the same GFP HDO transfected without Lipo lacked the ability to perform GFP gene silencing. This result confirms that we have successfully designed the GFP HDO to perform gene knockdown but that it lacks the ability for intracellular delivery. When the GFP HDO was conjugated with folic acid to result in GFP HDO-FA and then transfected with Lipo, the biological effect was superior (80% gene knock down). This result suggests that the centrally installed folic acid can increase the silencing activity of this new HDO. Recently Salim et al. reported on the gene-silencing activity of siRNA bearing folic acid modifications at different positions within the sense strand [39]. The authors showed that compared with siRNA modified on the termini, centrally modified folic acid siRNA enabled 80% gene silencing after treatment with 750 nM concentrations. Another report suggests that a centrally introduced mismatches in the structure of siRNA or double-stranded ASOs increases their biological efficacy and reduces toxicity [39,40]. A reasonable explanation for this effect is that the double-stranded oligonucleotides shall first dissociate, or the sense strand should be degraded by cellular enzymes, prior to hybridization to the target mRNA and performance of its mRNA knockdown action. On the other hand, the advanced parent oligonucleotide ASO5.2-FA when transfected with Lipo in the same concentrations showed a lower silencing effect (64%) compared with GFP HDO-FA. These results are in agreement with previous reports where double-stranded heteroduplex oligonucleotide showed higher knockdown compared with its single-stranded parent ASO [19,20,22,41].

Next, we tested the GFP HDOs conjugated only with folic acid, only with P1 or both biomolecules without Lipo transfection. Both GFP HDO-FA and GFP HDO-P1 showed intermediate gene silencing efficiencies by 30% and by 35%, respectively after 3000 nM treatment. Nevertheless, treatment with GFP HDO-FA-P1, bearing both attachments, the folic acid and the peptide sequence, at the same 3000 nM concentration led to 84% GFP gene knockdown. This improvement in the biological activity is significant and shows the importance of the additional effect of the attached folic acid and peptide. Fernandez et al. reported that the folic acid is a specific ligand for the folate receptor that is overexpressed in variety of cancer cells [33]. In addition, the benefit of a ligand–receptor interaction for the targeted intracellular delivery of oligonucleotide therapeutics is well known, [42] and the ability of folic acid to enhance the intracellular delivery through ligand-receptor endocytosis is studied [43]. Nevertheless, in the case of oligonucleotide therapeutics, recent reports highlight the importance of endosomal escape after delivery through endocytosis [44]. To perform its action after intracellular delivery, it is vital for the ASOs or siRNA to escape the endosomes and to be released in unrestricted form into the cytoplasm. Considering this, we designed the peptides, P1 and P2 to contain a cell-penetrating domain (CPD) (classical tat peptide) and an endosome escaping domain (EED) [45]. While the CPD favors crossing the cellular membrane, the EED plays a role in bursting the endosomal membrane and releasing the product into the cytoplasm. To our knowledge, there are no other reports in the literature that have examined the gene silencing activity of similar designs of advanced HDOs.

Finally, to check the biological action of the new HDO, we confirmed the gene-silencing activity of the endogenous *Bcl2* gene using real-time polymerase chain reaction (RT-PCR) in HeLa cells. Compared with the unconjugated Bcl2 HDO, the Bcl2 HDO-FA-P2 showed efficient by 44% *Bcl2* gene silencing. This result confirms that the folic acid and the P2 attachment improve intracellular delivery and the endosomal escape. Additionally, when we decorated the single-stranded parent ASO with both folic acid and P2 to yield Bcl2 ASO.1-FA-P2 it showed similar gene-silencing efficacy (by 46%) to the Bcl2 HDO-FA-P2, proving that the multi-labeled strategy might work for single-stranded ASOs. Since the multi-labeling process of single-stranded ASOs is challenging, further development of new chemical strategies will likely lead to simplified and cost-efficient synthesis that will inspire future systematic testing in vivo.

## 5. Conclusions

In conclusion, we reported an advanced design of multi-modified and multi-functionalized HDOs. The novel HDOs are decorated with an internally installed folic acid moiety and a terminal peptide sequence to achieve efficient gene silencing comparable to the efficacy of transfection with commercially available lipofectamine. Our results showed that a multi-functionalized HDO performed targeted intracellular delivery and by 84% GFP gene silencing. In addition, similarly designed HDOs could achieve effective *Bcl2* gene knockdown. We confirmed that rational sequence modification together with multi-labeling is important and aids the final performance of the HDOs and other single-stranded ASOs as well. We hope, this synthetic, multi-functional approach will provide an inspiration for combining other ligands, molecules and biomolecules. In addition, we suggest that this approach can be applied to other types of oligonucleotide therapeutics, such as siRNA.

## Data Availability

Not applicable.

## Supplementary Materials

The following supporting information can be downloaded at: https://www.mdpi.com/article/10.3390/biomedicines10092096/s1, Table S1: Oligonucleotide and peptide sequences used in this study; Scheme S1: Design of the HDO; Table S2: Information about the primers used for Real-time qRT-PCR. FW: Forward. RV: Reverse; Figure S1: Cell viability 48 h after transfection; Figure S2: Relative expression of the GFP in GFP-expressing HeLa cells; Figure S3: Relative expression of the GFP in GFP-expressing HeLa cells; Figure S4: Determination of the expression of folate receptor on the surface of the GFP HeLa cells; Figure S5: IC HPLC spectra; Figure S6: Maldi TOF spectra.
