# Supplementary material for: Multi-Functionalized Heteroduplex Antisense Oligonucleotides for Targeted Intracellular Delivery and Gene Silencing in HeLa Cells
Source: Biomedicines. 2022 Aug 27;10(9):2096. doi: 10.3390/biomedicines10092096 (PMC9495875; doi:10.3390/biomedicines10092096)
Supplement: Supplementary file 1 [file biomedicines-10-02096-s001.zip › biomedicines-1871129-SI.pdf]

Supporting Information

Multi-functionalized heteroduplex antisense oligonucleotides for targeted intracellular delivery and gene silencing in HeLa cells

Mauro Sousa de Almeida[1], Barbara Rothen-Rutishauser[1], Michael Mayer[2] and Maria Taskova[2*]

1. Bionano, Adolphe Merkle Institute, University of Fribourg, Chemin des Verdiers 4, 1700 Fribourg, Switzerland
2. Biophysics, Adolphe Merkle Institute, University of Fribourg, Chemin des Verdiers 4, 1700 Fribourg, Switzerland

Contents

1. Oligonucleotide, peptide and primer sequences 2
2. Cell viability after treatment with heteroduplex oligonucleotide (HDO)............ 43
3. GFP gene silencing in GFP expressing HeLa cells............ 53
4. Average number of folate receptors per cell 65
5. IC HPLC Spectra 76
6. Maldi TOF Spectra 109

1. Oligonucleotide, peptide and primer sequences

Table S1. Oligonucleotide and peptide sequences used in this study

Name	Sequence
ASO1	GTCGTGCTGCTTCATGTGGTC
ASO2	TCCTTGAAGAAGATGGTGCGC
ASO3	ATGTTGTGGCGGGTCTTGAAG
ASO4	TGCAGATGAACTTCAGGGTCA
**ASO5**	TGCCGGTGGTGCAGATGAACT
ASO6	TTGAAGAAGATGGTGCGCTCC
ASOscr	ACTGATGTCGCGGAGGTCTAG
ASO5.1	T*mG*mC* mC*G*G* T*G*G* T*G*C* A*G*A* T*G*mA* mA*mC*T
ASO5.2	T*mG*mC*m C*G*G*T*G*G*T*G*C*A*G*A* /i5OctdU/*G*mA* mA*mC*T
ASOss	rArUrC rUrGrC rArCrC rArCrC rGrGrC rA/3ThioMC3-D/
Bcl2 ASO	A*mU*mG*mA*C*T*G*C*T*A*C*G*A*A*G*T*T*mC*mU*mC*C
Bcl2 ASO.1	/5AmMC6/A*mU* mG*mA*C* T*G*C* T*A*C* G*A*A* G*/i5OctdU/*T* mC*mU*mC* C
Bcl2ss	CTT CGT AGC AGT CAT/35OctdU/
Bcl2scr	ACA CTA TAC GTT CCA CGT GTG
P1	{Mpa}GFWFG{PEG6}RKKRRQRRR
P2	{Lys(N3)} GFWFG{PEG6}RKKRRQRRR

- * is indication for a phosphorothioate backbone; m is 2’OMe modification; i5OctdU is an internal 5-Octadiynyl dU modification; 3ThioMC3-D is a 3' Thiol Modifier C3 S-S modification; 5AmMC6 is a 5' Amino Modifier C6 modification; Mpa is a a terminal 3-mercaptopropyl; N3 is terminal azide.

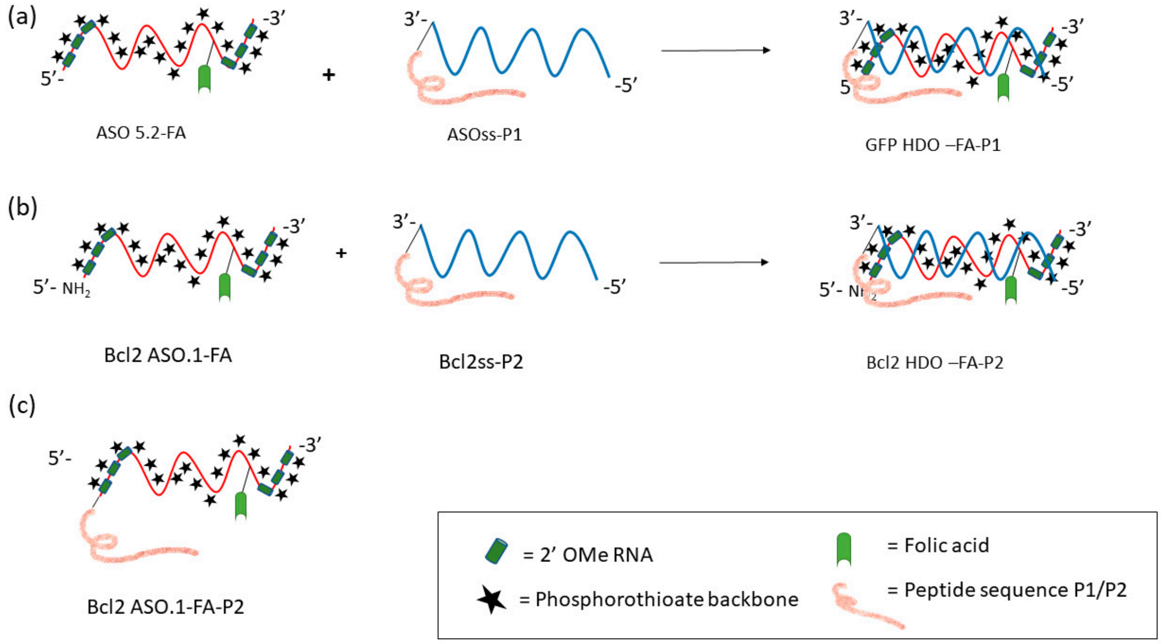

Scheme S1. Design of the HDO; (a) the GFP HDO-FA-P1 consists of the ASO 5.2 conjugated with FA and the ASOss conjugated with P1 followed by hybridization; (b) the Bcl2 HDO-FA-P2 consists of the Bcl2 ASO.1 conjugated with folic acid and the Bcl2ss conjugated with P2 followed by hybridization; (c) the Bcl2 ASO.1-FA-P2 is conjugated with FA followed by conjugation with P2. The sequences of the oligonucleotides and the peptides are presented in Table S1. The conjugation chemical strategies are presented in Scheme 1. FA is folic acid and P1/P2 are the peptides.

Table S2. Information about the primers used for Real-time qRT-PCR. FW: Forward. RV: Reverse.

Gene symbol	*Gene name*	*Sequence (5'->3')*	*Product length*	*Efficiency*
GAPDH	Glyceraldehyde-3-phosphate dehydrogenase	FW: TTGCTGATGGTGCTGTCTCC	147	1,97
		RV: CAGTGGTAGCTGTTTAACCTTGC		
YWHAZ	Tyrosine 3-monooxygenase/tryptophan 5-monooxygenase activation protein zeta	FW: GAGACAGAGCGAATCGTCACC	120	1,94
		RV: TGTCGATCAGCAGAAGAATCTGG		
BCL2	BCL2 apoptosis regulator	FW: CTGGTGGGAGCTTGCATCAC	150	1,88
		RV: ACAGCCTGCAGCTTTGTTTC		

2. Cell viability after treatment with heteroduplex oligonucleotide (HDO)

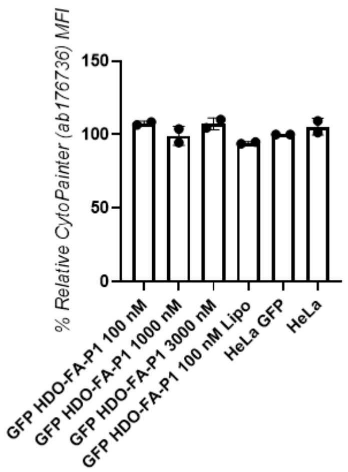

Figure S1. Cell viability 48 h after transfection. The cell proliferation was assessed using CytoPainter cell proliferation assay. The mean fluorescence intensity (MFI) was measured by flow cytometry. Error bars indicate standard deviation (SD) of at least two independent replicates.

3. GFP gene silencing in GFP expressing HeLa cells

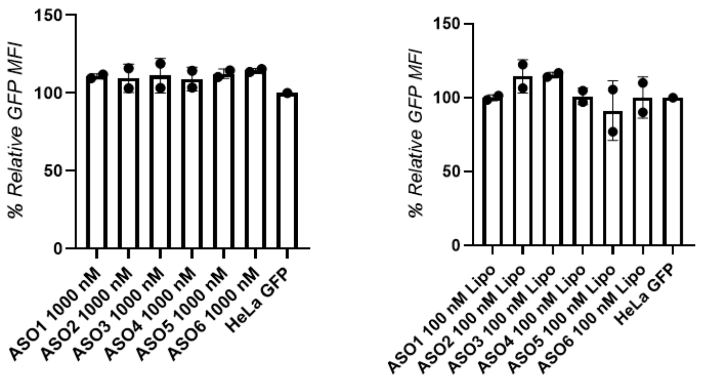

Figure S2. Relative expression of the GFP in GFP expression HeLa cells. Transfection was performed with lipofectamine (Lipo) or without. GFP expression was assessed measuring the median fluorescence intensity (MFI) 48 h post transfection by flow cytometry. Error bars indicate standard deviation (SD) of at least two independent biological replicates.

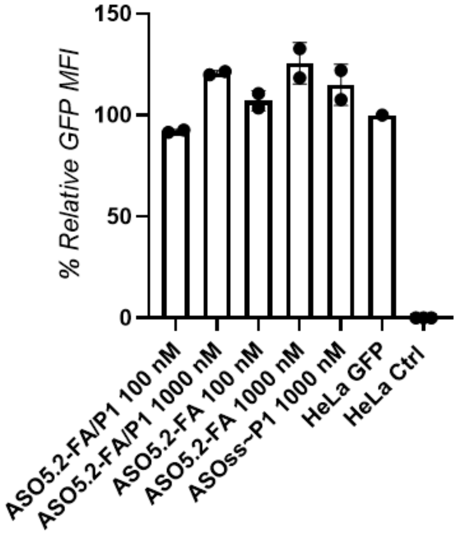

Figure S3. Relative expression of the GFP in GFP expression HeLa cells. Transfection was performed without lipofectamine. FA is folic acid; P1 is peptide 1. GFP expression was assessed measuring the median fluorescence intensity (MFI) 48 h post transfection on an incucyte-imaging microscope. Error bars indicate standard deviation (SD) of at least two independent biological replicates.

4. Average number of folate receptors per cell

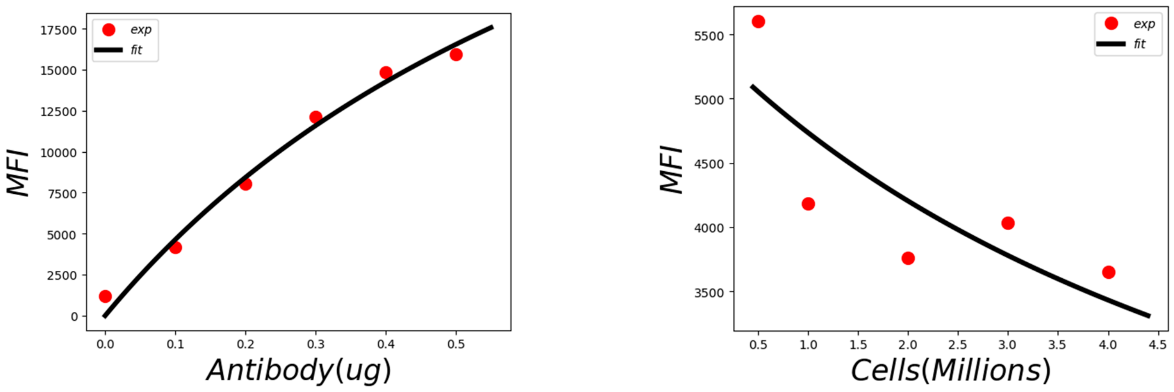

Figure S4. Determination of the expression of folate receptor on the surface of the GFP HeLa cells by titration against increasing amount of a) antibody or against increasing number of b) cells added. Median fluorescence intensity (MFI) was obtained using flow cytometry measurements (red circles). The fitted curves were obtained by fitting the experimental data with the equations bellow.

We used previously described method to calculate the average number of folate receptors on each HeLa cell used in the project. Briefly, we first incubate a fixed number of cells (0.5 million) per condition with increasing concentration of a fluorescent antibody, APC anti-human folate receptors alpha and beta (0 – 0.5 µg) which specifically bind to the folate receptor. We measured the median fluorescence intensity (MFI) resulting from the bound antibodies by FACS. A binding equation:

$$FI = \frac{FI_{MAX} \times [Ab]}{K_{Ab} + [Ab]}$$

was fitted to the experimental values. In the equation, [*Ab*] is the concentration of antibody in each condition, *FI* is the measured median fluorescence, FI_{MAX} is the maximal fluoresce intensity measured by the maximal binding of antibody and K_{AB} is the concentration of antibody that binds to one-half of folate receptors on the GFP HeLa cell surface.

Next, we incubated fixed concentration of antibody, APC anti-human folate receptors alpha and beta (0.1 µg) per condition with increasing number of cells (0.5 – 4 million) and we measured the median fluorescence intensity. The measured values were fitted to a depletion equation:

$$FI = \frac{FI_{INIT} \times K_C}{K_C + [Cells]}$$

where FI_{INIT} is the initial fluorescence intensity determined by the maximal binding of antibody, [*Cells*] is the number of cells in each reaction, and *Kc* is the number of cells that depletes one-half of the maximal binding. By fitting the experimental values, we found K_{AB} to be 0.8 ug per condition and *Kc* to be 6.9 million cells per condition. The calculated number of folate receptor per one GFP HeLa cell is 1.7 million.

5. IC HPLC Spectra

(A)

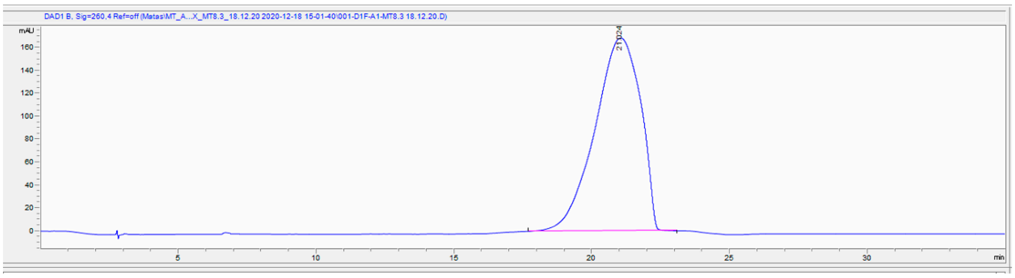

(B)

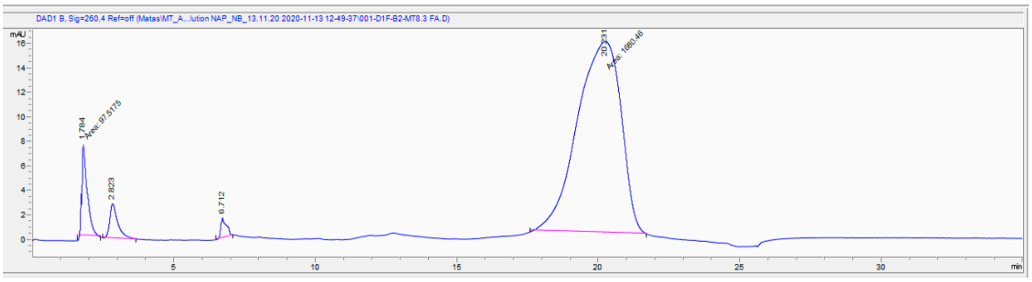

(C)

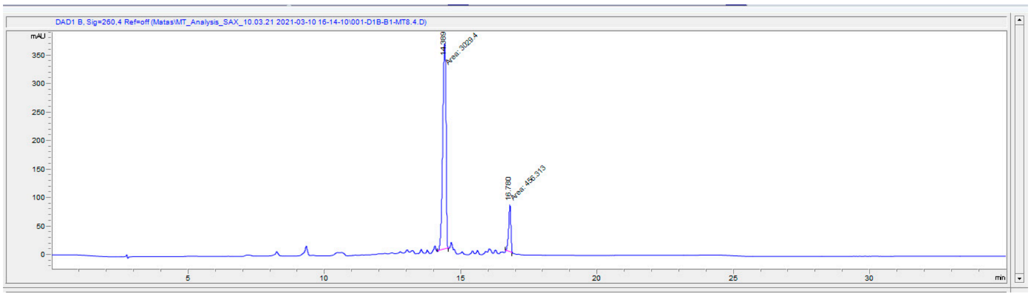

(D)

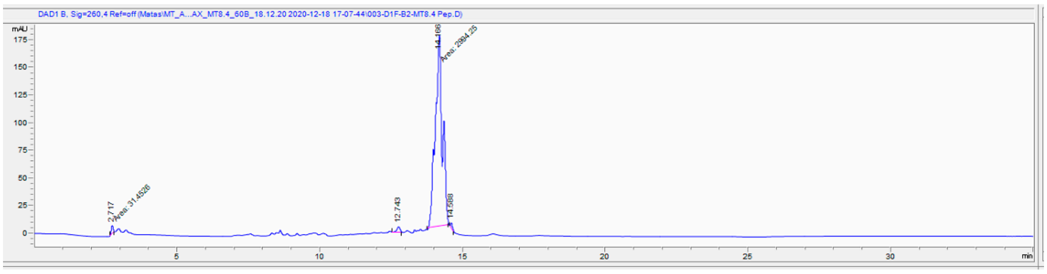

(E)

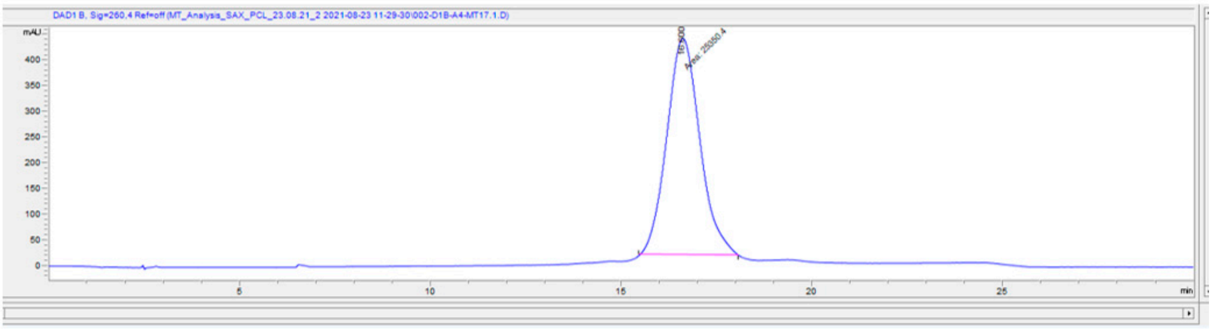
DAD1 B, Sig=260,4 Ref=off (MT_Analysis_SAX_PCL_23.08.21_2 2021-08-23 11-29-30\002-D1B-A4-MT17.1.D)
mAU
Area: 25350.4
min

(F)

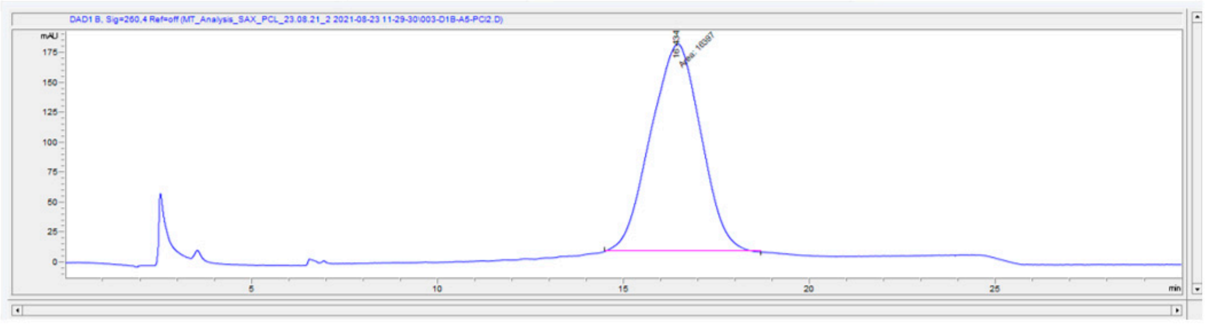
DAD1 B, Sig=260,4 Ref=off (MT_Analysis_SAX_PCL_23.08.21_2 2021-08-23 11-29-30\003-D1B-A5-PCl2.D)
mAU
Area: 10397
min

(G)

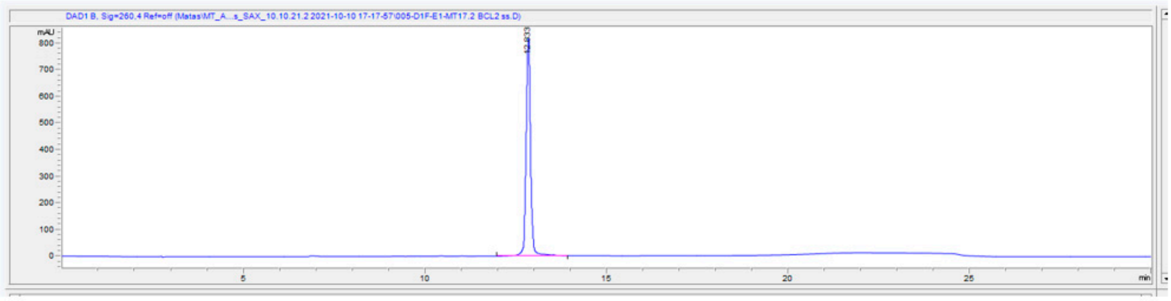
DAD1 B, Sig=260,4 Ref=off (Matas\MT_A...s_SAX_10.10.21.2 2021-10-10 17-17-57\005-D1F-E1-MT17.2 BCL2 ss.D)
mAU
min

(H)

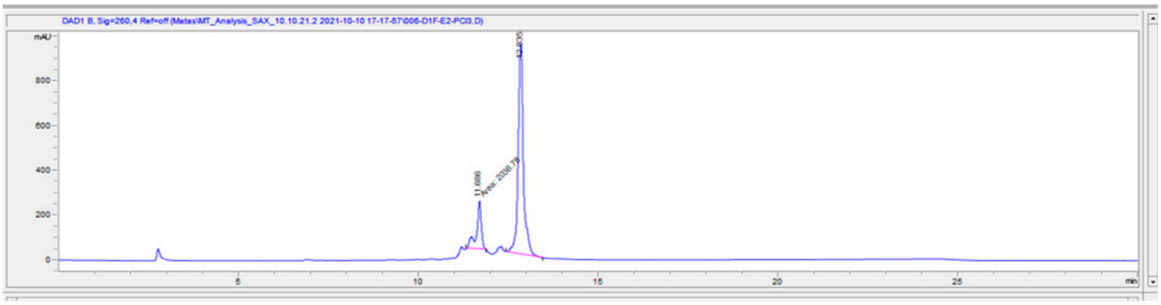
DAD1 B, Sig=260,4 Ref=off (Matas\MT_Analysis_SAX_10.10.21.2 2021-10-10 17-17-57\006-D1F-E2-PCl3.D)
mAU
11.686
Area: 2036.78
min

(I)

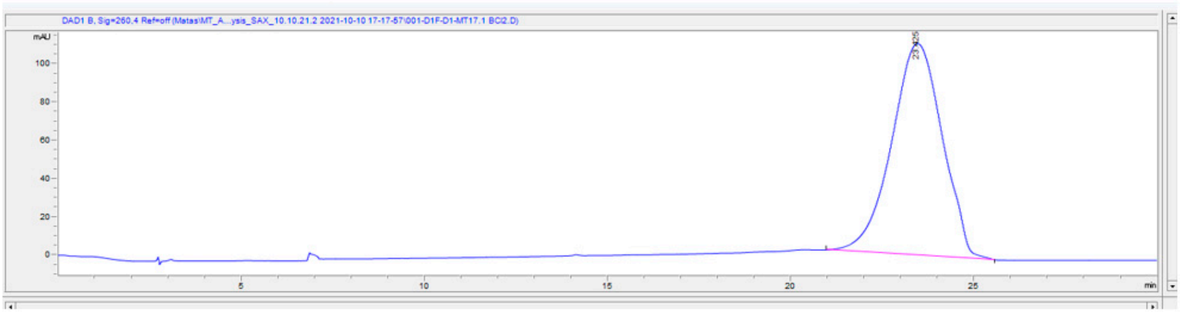

(J)

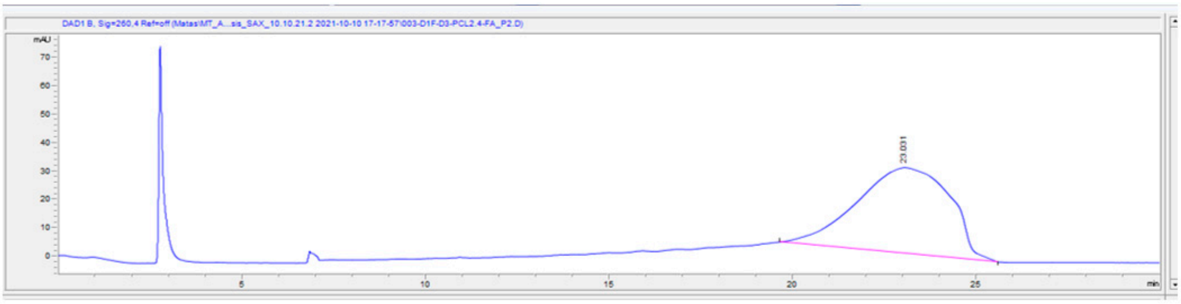

Figure S5. IC HPLC spectra of the (A) unlabeled antisense oligonucleotide, ASO 5.2 (RT: 21.024, 100%); (B) antisense oligonucleotide labeled with folic acid, ASO 5.2-FA (RT: 20.231, 90.5%); (C) unlabeled sense oligonucleotide, ASOss (RT: 14.541, 85.0%); (D) sense oligonucleotide labeled with P1, ASOss-P1 (RT: 14.166, 96.9%); (E) Bcl2 unlabeled antisense oligonucleotide, Bcl2 ASO.1 (RT:16.60, 100%); (F) Bcl2 antisense oligonucleotide labeled with folic acid, Bcl2 ASO.1-FA (RT:16.43, 100%); (G) Bcl2 unlabeled sense oligonucleotide, Bcl2ss (RT:12.8, 100%); (H) Bcl2 sense oligonucleotide labeled with P2, Bcl2ss-P2 (RT:12.8, 82.4%); (I) Bcl2 unlabeled antisense oligonucleotide, Bcl2 ASO.1 (RT:23.4, 100%); (J) Bcl2 antisense oligonucleotide labeled with folic acid and P2, Bcl2 ASO.1-FA-P2 (RT:23.03, 100%).

.

6. Maldi TOF Spectra

(A)

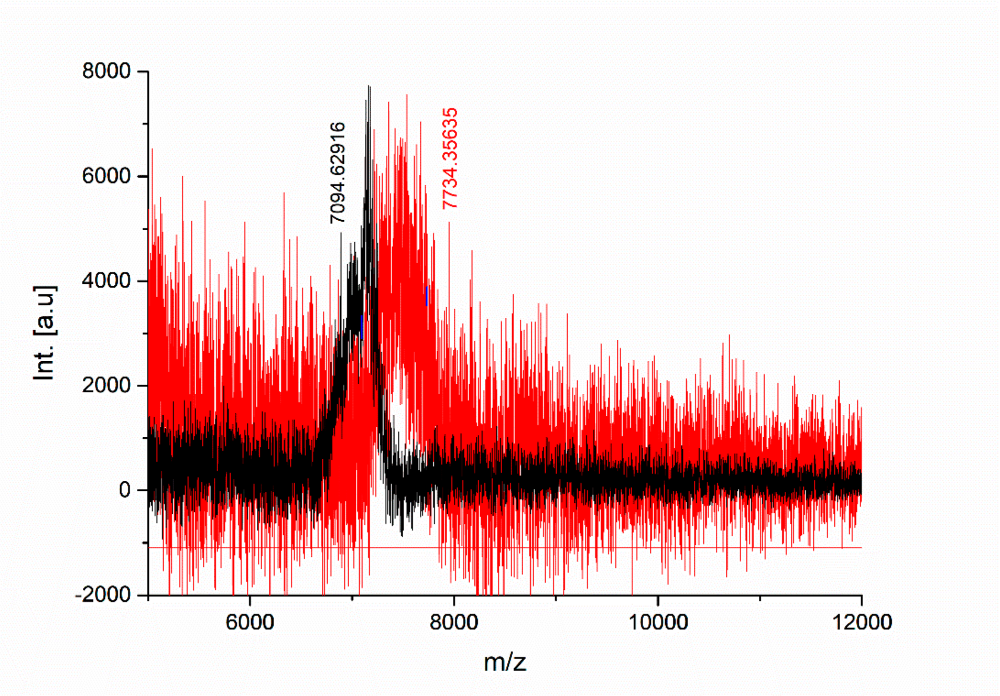

(B)

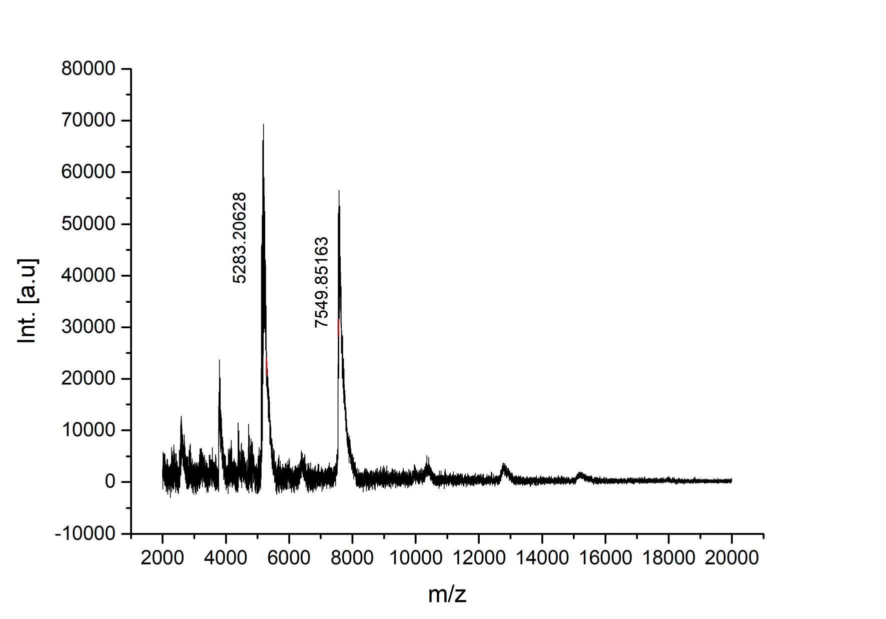

(C)

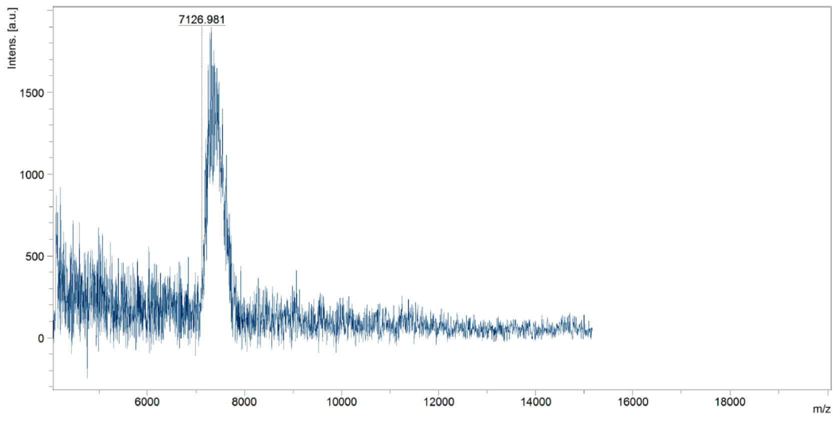

7126.981
Intens. [a.u.]
1500
1000
500
0
6000
8000
10000
12000
14000
16000
18000
m/z

(D)

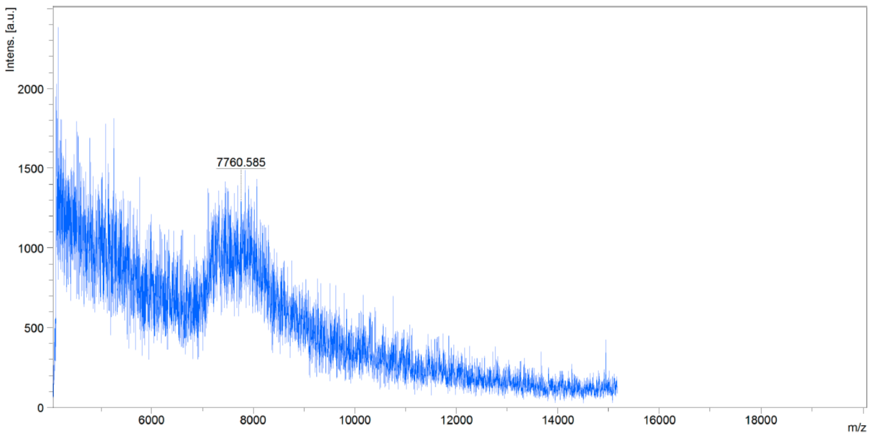

7760.585
Intens. [a.u.]
2000
1500
1000
500
0
6000
8000
10000
12000
14000
16000
18000
m/z

(E)

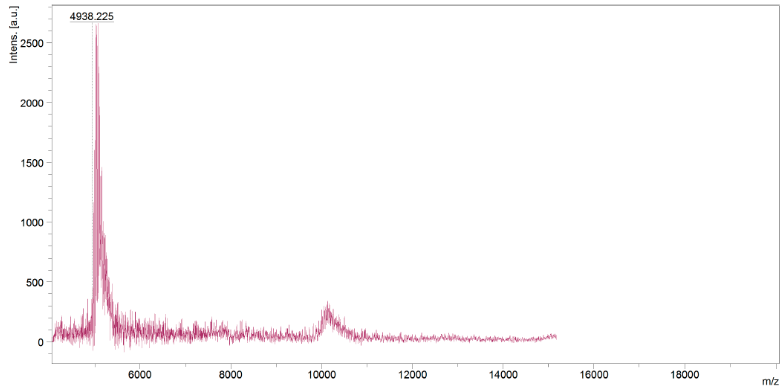

(F)

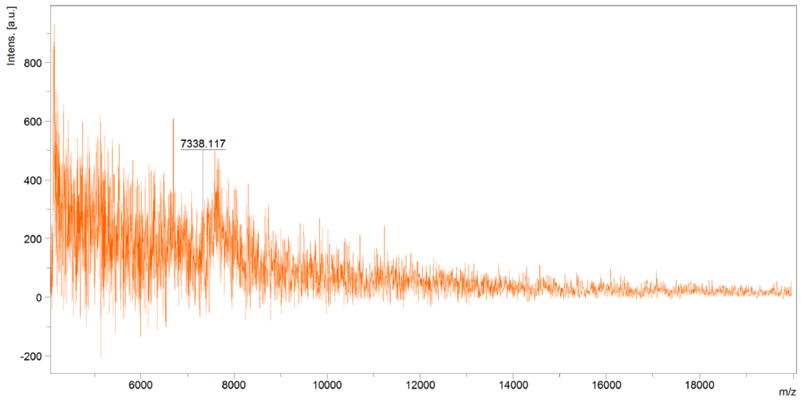

Figure S6. Maldi TOF spectra. (A) ASO 5.2 (Mw=7094.6 g/mol / Mw calc.=7093.8 g) / ASO 5.2-FA (Mw=7734.3 g/mol / Mw calc.=7735.4 g/mol); (B) ASOss-P1 (Mw=7549.85 g/mol / Mw calc.=7550 g/mol); (C) Bcl2 ASO.1 (Mw=7126.9/Mw calc.=7123.2); (D) Bcl2 ASO.1-FA (Mw=7760.5/Mw calc.=7764.0); (E) Bcl2ss (Mw=4938.2/Mw calc.=4937.0); (F) Bcl2ss-P2 (Mw=7338.1/Mw calc.=7324.0). The sequences of the oligonucleotides are shown in Table S1. Mw calc. is the theoretically calculated Mw.
